# Draft Genome Sequence of *Arthrobacter* sp. Strain 260, Isolated from a Uranium Tailings Management Facility in Northern Saskatchewan, Canada

**DOI:** 10.1128/MRA.00360-21

**Published:** 2021-07-01

**Authors:** Alexander A. Grigoryan, Viorica F. Bondici, Yuriy Kryachko, Nurul H. Khan, John R. Lawrence, Sanjit Singh, Gideon M. Wolfaardt, Niradha Withana Gamage, Deeksha Shetty, Darren R. Korber

**Affiliations:** a Department of Food and Bioproduct Sciences, University of Saskatchewan, Saskatoon, Saskatchewan, Canada; b Canadian Light Source, Saskatoon, Saskatchewan, Canada; c Saudi Aramco, Dhahran, Saudi Arabia; d Lallemand Plant Care, Lallemand Inc., Saskatoon, Saskatchewan, Canada; e Environment Canada, Saskatoon, Saskatchewan, Canada; f Department of Chemistry and Biology, Ryerson University, Toronto, Ontario, Canada; g Department of Microbiology, Stellenbosch University, Stellenbosch, South Africa; Loyola University Chicago

## Abstract

The 3.9-Mbp draft genome sequence of *Arthrobacter* sp. strain 260, which was isolated from a uranium tailings management facility, is reported. The sequence may help determine the bioremediation potential of this strain and facilitate further research aimed at a better understanding of the hypertolerance of this genus to extreme conditions.

## ANNOUNCEMENT

*Arthrobacter* spp. are aerobic microorganisms that are known to be capable of living under extreme conditions, particularly in environments contaminated with compounds such as arsenites (1) and selenates and selenites (2) and in the presence of heavy metals, including radionuclides (3–7). In a number of studies (e.g., references 4, 6, and 7), *Arthrobacter* spp. were found to abound in uranium-rich environments. To the best of our knowledge, however, only one whole-genome sequencing project was previously dedicated to an *Arthrobacter* sp. from a uranium-rich environment (6).

Here, we report the draft genome sequence of *Arthrobacter* sp. strain 260, which was isolated from a uranium tailings management facility in Key Lake in northern Saskatchewan, Canada. Strain 260 (isolate code AET35A) originated from a tailings sample collected at a 35-m depth from the tailings-water interface (7). To isolate the microorganism, 0.2 g of tailings was suspended in 1 ml of sterile Tris-EDTA (TE) buffer (pH 8), plated on 5% tryptic soy agar (TSA), and incubated aerobically at 5°C for 3 weeks. Following isolation, colonies were subcultured three times. The pure culture has been stored in 15% glycerol/5% tryptic soy broth (TSB) at −80°C. A DNA extraction kit (Qiagen, Germantown, MD, USA) was used to extract DNA from glycerol stock cells, which were regrown on 5% TSA. Genus-level identification of the isolate was performed through 16S rRNA and *cpn60* gene amplification and sequencing, as described by Bondici et al. (7).

Whole-genome sequencing was carried out using a MiSeq sequencing platform (Illumina, Inc., San Diego, CA, USA). The genome library was constructed using the Nextera XT library preparation kit and the MiSeq reagent 300-cycle v2 kit (Illumina) following the manufacturer’s instructions. As a result of sequencing, 1,856,606 paired-end reads (541 Mbp) were generated.

Sequence read error correction, quality trimming, contig assembly, misassembly correction, and scaffolding were performed using the SPAdes assembler v3.12.0 (8), with k-mer sizes of 21, 33, and 55. The genome consists of 193 contigs (*N*_50_ value of 221,532 bp) and is 3,916,467 bp long, excluding gaps; the genome coverage is 82×, and the G+C content is 63.8%.

Annotation of the genome was performed using the NCBI Prokaryotic Genome Annotation Pipeline (PGAP) v4.11 (9). As a result of annotation, 3,647 protein-coding sequences and 57 RNAs were identified in the genome.

### Data availability.

This whole-genome shotgun project has been deposited in DDBJ/ENA/GenBank under the accession number JABFOE000000000. Raw data were deposited in the SRA under the accession number SRR11789134 (BioProject number PRJNA631432).
